# The Role of Moderating Variables on BOLD fMRI Response During Semantic Verbal Fluency and Finger Tapping in Active and Educated Healthy Seniors

**DOI:** 10.3389/fnhum.2020.00203

**Published:** 2020-06-05

**Authors:** Claudia Rodríguez-Aranda, Susana A. Castro-Chavira, Ragna Espenes, Fernando A. Barrios, Knut Waterloo, Torgil R. Vangberg

**Affiliations:** ^1^Department of Psychology, Faculty of Health Sciences, UiT The Arctic University of Norway, Tromsø, Norway; ^2^Instituto de Neurobiología, Universidad Nacional Autónoma de México, Querétaro, Mexico; ^3^Department of Neurology, University Hospital of North Norway, Tromsø, Norway; ^4^Department of Radiology and Nuclear Medicine, University Hospital of North Norway, Tromsø, Norway; ^5^Department of Clinical Medicine, UiT The Arctic University of Norway, Tromsø, Norway

**Keywords:** semantic verbal fluency, fMRI, demographic variables, proxies, cognitive reserve, covert study, finger tapping

## Abstract

Semantic verbal fluency is among the most employed tasks in cognitive aging research and substantial work is devoted to understanding the underlying mechanisms behind age-related differences at the neural and behavioral levels. The present investigation aimed to evaluate the role of moderating variables, such as age, sex, MMSE, and proxies of cognitive reserve (CR) on the hemodynamic response evoked by semantic verbal fluency in healthy young and healthy older adults. So far, no study has been conducted to this end. To elucidate the exclusive effect of the mentioned variables on brain activation during semantic fluency, finger tapping was included as a control task. Results showed that disregarding adjustments for age, older adults displayed important parietal activations during semantic fluency as well as during finger-tapping. Specifically, the anterior intra-parietal sulcus (IPS) and left inferior parietal lobule (IPL) were areas activated in both tasks in the older group. Younger adults, only displayed parietal activations related to age and sex when these demographics were employed as predictors. Concerning proxies of CR in semantic fluency, the only vocabulary was an important moderator in both age groups. Higher vocabulary scores were associated with lesser activation in occipital areas. Education did not show significant correlations with brain activity during semantic fluency in any of the groups. However, both CR proxies were significantly correlated to brain activations of older adults during finger tapping. Specifically, vocabulary was associated with frontal regions, while education correlated with parietal lobe and cingulate gyrus. Finally, the effects of MMSE were mostly observed on brain activation of older adults in both tasks. These findings demonstrate that the effects of moderating variables on shaping brain activation are intricate and not exclusive of complex verbal tasks. Thus, before adjusting for “nuisance variables,” their importance needs to be established. This is especially true for samples including older adults for whom a motor task may be a demanding operation due to normal age-related processes of dedifferentiation.

## Introduction

Verbal fluency tasks are among the neuropsychological tests most widely employed in both experimental and clinical aging studies. These tasks assess the capacity to accurately and quickly produce words matching either a letter of the alphabet (phonemic fluency) or a semantic category (semantic fluency) within a limited time. Of these two variants, the semantic fluency is of particular interest for aging research, since generating words according to a determined category has shown larger age-related differences than producing words according to an initial letter. Although there is no clear consensus as for why semantic fluency declines more in aging than phonemic fluency, it is proposed that the reason relies on its dependency on semantic memory (Gardini et al., 2013), which deteriorates dramatically in pathological aging (Gainotti et al., 2014). Probably, for this reason, semantic fluency is recurrently used as one of the most discriminatory tests for the early diagnosis of dementia (Heun et al., 1998; Mitchell and Malladi, 2010).

Because semantic verbal fluency is widely employed in aging studies, it is important to understand the moderating factors that help maintain semantic fluency. The literature proposes that semantic fluency is not only influenced by increasing age (e.g., Tallberg et al., 2008; Stolwyk et al., 2015) but also by sex (Capitani et al., 1999; Lanting et al., 2009), education (da Silva et al., 2004; Tallberg et al., 2008), vocabulary (Shao et al., 2014) and global mental status (Obeso et al., 2012). The importance of these factors has been evaluated at the behavioral level (van Hooren et al., 2007), while their relationship with brain activity is still largely unknown. As a rule, demographics in neuroscientific research are used either to obtain balanced study designs or as “nuisance variables” to minimize their influence on the variability of data; albeit, demographics are usually of marginal importance for the purpose of the investigation at hand. However, some studies have addressed the impact of demographics on brain structure and function as well as on cognitive ability, showing the importance of the ubiquitous effects of demographics on cognition and brain measures. A compelling example of these studies demonstrated that when cognitive functions are measured in older adults, adjusting for age is not advisable as it decreases sensitivity to age-differences in brain structure (Mungas et al., 2009). Moreover, the same study disclosed the importance of adjusting data for ethnicity in aging research.

As for semantic fluency, to the best of our knowledge, only two studies have evaluated the effect of restricted demographics on the neural activation of semantic fluency. The first one, conducted by Nagels et al. (2012), assessed the influence of vocabulary and age in healthy young and middle-aged participants during an overt fMRI paradigm. The second one, also an overt fMRI study (Marsolais et al., 2015), tested the effects of age on the brain activity of healthy young and older participants. In the former, the authors found significant correlations between age and prefrontal cortex activation and between vocabulary and right superior temporal gyrus activation. In the latter, increased activation in bilateral postcentral gyri was found for the young group while decreased activation in left inferior frontal gyrus and superior parietal lobule (SPL) occurred in the older group.

Although these investigations represent the first step to better appraise the role of demographics on brain activation during semantic fluency in healthy individuals, the issue is far from settled. In the present study, we expand this line of research not only by further addressing the impact of common demographic variables on brain activity during semantic fluency but also by evaluating the role of those variables converging with the concept of “cognitive reserve” (CR).

### Demographics and Variables Reflecting Cognitive Reserve (CR)

Some demographic variables have been used as indicators of CR. Since CR refers to the protective aspect that life experiences and events offer when cognitive and/or brain insults occur in aging (Jefferson et al., 2011), the effects of variables reflecting CR are noteworthy. For example, the existence of age-related brain degeneration while cognitive capacity remains unchanged is not uncommon. The explanation can be a large CR promoting compensatory mechanisms. In this context, it is relevant to understand which factors are associated with compensatory brain mechanisms. For instance, it would be relevant to know whether sex or education modulates cerebral activation during semantic fluency execution. The issue is of central interest for healthy older adults showing behavioral outcomes similar to younger adults. The approach is not a new one. Scarmeas et al. (2003) examined how a set of variables reflecting CR (i.e., education, and vocabulary among others) correlated with the neural response evoked by a memory task. The authors’ rationale was that if there is compensation due to CR in aging, the effect should be reflected not only in the behavioral outcome but also in the neural response of older adults. The authors confirmed that CR mediated the neural activation in younger and older adults differently, which was interpreted as a modulatory effect of CR variables on compensatory brain reorganization of older participants. In the context of the present investigation, CR variables need to be examined to provide a better insight into factors that preserve semantic fluency in healthy aging.

### Selection of Variables Evaluated in the Present Study

In the present study, we selected a set of variables relevant to semantic fluency. To begin with, we selected education and verbal knowledge. Education is by far the most used indicator of CR (Jones et al., 2011), while vocabulary is an inherent requisite for verbal abilities (Verhaeghen, 2003). Moreover, age and sex are typical demographics that need to be controlled, especially since these variables are interrelated with years of education and vocabulary (Jones et al., 2011), which are two of the relevant covariates in this study. In the case of education, it is often associated with a person’s age and sex, since the educational system largely predetermines the average level of formal schooling in a given cohort. This is especially true for older adults who were subject to compulsory public education in Europe and the Nordic countries in the middle of the twentieth century (Blossing et al., 2014). Several studies have demonstrated that such reforms had a strong positive impact on the mental abilities of men but not on women (Banks and Mazzonna, 2012). The reasons are mainly related to lifestyles of that generation that endorse better working conditions for educated men, while women remained at home or with minimal participation in the labor force. The generation affected by the educational reforms conforms today to the older adult population in their 70s, such as older participants in our study. As for verbal knowledge, the same inferences are true as it is well documented that lexical ability is related to formal schooling (Verhaeghen, 2003). The implications of elucidating whether the mentioned variables modulate brain activation during semantic fluency are important to clarify. Such information would promote strategies like cognitive or physical training that increase neuroplasticity (Park and Bischkopf, 2013). Hence, CR can also be enhanced in specific subpopulations of healthy older adults (e.g., women) and which may be observable in semantic verbal fluency.

However, the issue of interrelationship poses an immediate implication: If these variables are interrelated, it seems logical to expect that their modulating effect on brain activation would also be, at least to some extent, overlapping in similar brain regions. Unfortunately, no previous research exists that gives us a base or a rejection of this initial premise. For this reason, our working hypothesis is that possible protective effects of various demographics should converge in similar brain areas of older adults, which may differ from younger participants. Finally, we decided to add another variable relevant to cognitive aging, namely global mental status as measured by the MMSE. As this test is of clinical interest in the detection and progression of pathological aging and since it is associated with semantic fluency (Obeso et al., 2012), we decided to explore its relationship with brain activation during semantic fluency performance.

Therefore, the present study aims to evaluate the role of age, sex, education vocabulary, and MMSE on the neural activation during the performance of semantic fluency in young and healthy older adults. In contrast to the earlier studies addressing the same question, we applied a covert fMRI paradigm. Since overt and covert fMRI designs reliably activate the same brain areas during verbal fluency performance (Costafreda et al., 2006) and both detect consistently lateralized brain areas (Partovi et al., 2012; Gutierrez-Sigut et al., 2015), this approach should be complementary to previous studies that have used overt paradigms. Finally, to understand whether the effects of the moderators are exclusive to semantic fluency we also compared the moderating effects of demographics to a simple motor task, namely finger tapping. Based on previous literature (Wu and Hallett, 2005), we did not expect to detect important group differences on the effects of the selected variables on finger tapping.

## Materials and Methods

### Participants

Fifteen young (22–36 years old; eight men) and seventeen healthy older (65–76 years old; nine men) right-handed, native Norwegian speakers were recruited for the study. The older participants were physically active and engaged in regular social and recreational activities. All the participants underwent screening with the Mini-Mental State Examination (MMSE) (Folstein et al., 1975), and the Beck Depression Inventory (BDI; Beck et al., 1996) to rule out pathological cognitive deterioration and depression, respectively. Recruitment of the young group was conducted through advertisements and flyers at the university campus. Recruitment of the older group was performed through advertisements and oral information at senior citizens’ centers, local organizations promoting social and intellectual activities for older persons (Senioruniversitet i Tromsø), and sport-clubs for healthy older adults. A short initial interview was conducted to gather demographic and health information.

Inclusion criteria comprised of being in good health, taking no medication affecting the nervous system, being right-handed, having Norwegian as mother tongue, and age between 20 and 40 years for younger participants and over 60 years for older participants. To assure the inclusion of healthy participants, the Norwegian version (Loge et al., 1998) of the RAND Short Form 36 (SF-36) was administered. Additionally, all the participants were free of psychiatric or neurological illness, tumors, drug or alcohol abuse. A neuroradiologist screened the MR images for major pathologies such as infarctions or tumors. All participants were aware that participation in the study was voluntary and provided signed, informed consent before the interview, and testing. Participants were aware that they could quit the study at any point if they so choose and without further explanation. The study was approved by the local Research Ethics Committee.

### Procedure and Neuropsychological Evaluation

#### Session 1

In the first session, participants met at the Department of Psychology for an initial interview. In this interview, participants were asked to report the exact number of years they attended formal school as well as their highest academic degree achieved. The total number of formal years at school accounted for education in the analyses.

##### Background Tests

Participants were evaluated with a neuropsychological test battery to acquire a comprehensive cognitive profile for each group. This strategy enabled us to better understand possible age-related differences in verbal fluency in respect of group differences in other cognitive domains. The battery included the Digits Span forward and backward of the WAIS-R (Wechsler, 1981); the Logical Memory Test I and II from the Wechsler Memory Scale, 3rd edition (Wechsler, 1997); the Norwegian version of the Stroop test (Golden, 1978); the Purdue Pegboard Test (Lafayette Instrument Model 32020; Tiffin, 1968); and Finger tapping test (Reitan and Wolfson, 1985).

##### Main Study Tests

The vocabulary subtest of the WAIS-R (Wechsler, 1981) was selected to measure lexical abilities and accounted for vocabulary scores in the fMRI analyses. Verbal fluency was assessed with a procedure developed in our laboratory (for a complete description see Rodríguez-Aranda and Jakobsen, 2011). In brief, this procedure includes a computerized adaptation of the verbal fluency task presented with the E-prime computer program (Psychology Software Tools, Pittsburgh, PA, USA). Participants were required to sit 50 cm away in front of a PC monitor and wearing a headset microphone. Oral and written instructions were given to the participants before the assessment. The participants’ responses were registered with the Computerized Speech Lab equipment (CSL 4500, Kaypentax). In the behavioral session, both types of verbal fluency (i.e., semantic, and phonemic) were assessed to obtain a general measure of verbal fluency performance. For phonemic fluency, the letters “F,” “A” and “S” were used. For semantic fluency, the categories employed were “Animals,” “Fruits” and “Professions.” All stimuli were shown on the screen for 1 min and participants were asked to start generating words as soon as a letter or category appeared on the screen. The number of correct words per category, errors, and perseverations were registered and scored afterward.

#### Session 2

**Figure 1:**
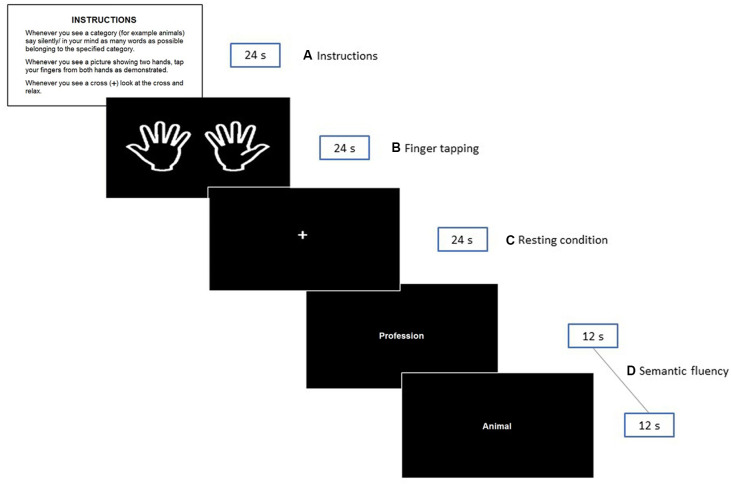
Illustration of the fMRI paradigm shown to the participants during the experiment. The instructions presented in Norwegian to the participants were: INSTRUKSJONER. Når du ser en kategori (f.eks. dyr) tenker du på/ sier inni deg så mange ord som mulig som tilhører den angitte kategorien. Når du ser «to hender»—beveger du fingrene som anvist. Når du ser et kryss (+) på skjermen—ser du på krysset og slapper av.

The second session was scheduled within 1 week after session 1 at the Department of Radiology, University Hospital of North Norway. Participants were required to arrive well ahead of the scanning time to obtain a detailed explanation about the experimental protocol and to be trained for the fMRI session. Four stimuli cards printed on paper were used for explanatory purposes (see Figure 1). These cards were replicas of some of the stimuli presented in the scanner during the experimental situation.

Participants were trained to respond adequately to each card by using a computer. They were not scanned until it was evident to the examiner that the participant had completed the paradigm according to the instructions provided. An alternative set of categories was used to avoid priming effects. After having executed the training paradigm, the participants were instructed to repeat the task, but this time quietly inside their minds and without moving their lips. To guarantee that all participants had understood the instructions, the training was first performed with a verbal response and then silently before entering the scanner. Participants were then informed and prepared to execute the paradigm silently during the scanning session. The silent execution of the semantic verbal fluency task was used to prevent head movement associated with speech production, thus ruling out this bias in the fMRI.

### fMRI Task Paradigm

A silent semantic verbal fluency paradigm consisting of a block design with three conditions was created. Epochs of 24 s were allowed for the silent generation of semantic examples matching specific categories. The following six categories were used: animals, fruits, professions, supermarket items, body parts, and countries. These categories were selected based on previous studies of verbal fluency (e.g., Hirshorn and Thompson-Schill, 2006; Kormi-Nouri et al., 2008) and on pilot data from our laboratory (see Supplementary Table S1). Notably, the selected categories are straight forward classes, considered “easy categories” (Mayr and Kliegl, 2000) where no age-related differences have been reported (Sauzéon et al., 2011). On each epoch, a pair of categories were presented. The word for each category was presented in the middle of the screen for 12 s. This adaptation assures the active silent production of categories, as it has been proven that word production is higher during the first 30 s (Crowe, 1998) and that the first 5–10 s are critical for word generation in older adults (Lee et al., 2015). Periods of semantic fluency production were interleaved with a finger tapping task that was performed simultaneously with both hands. As mentioned in the introduction, the control task served as a contrast situation for semantic epochs to better understand whether the effects of the demographics and other selected variables were exclusively related to semantic fluency. Participants were instructed to simultaneously tap their index fingers against the thumbs of both hands each time a picture of two extended hands appeared on the screen (from left to right; see the 2nd rectangle in Figure 1). They were asked to perform sequential finger tapping at a self-selected pace until the picture is no longer visible. Finally, semantic and finger tapping epochs were alternated with a white cross in the center, denoting resting intervals.

Thus, each condition had a duration of 24 s and was repeated six times in the following order: Finger tapping > resting condition > semantic fluency. The six possible categories of semantic fluency were pseudorandomly (i.e., the same categories were not presented consecutively) presented at each epoch.

### fMRI Acquisition

Brain images were acquired in a Phillips Magnetom 1.5 T scanner. T1 structural images were used to discard pathologic radiological findings: sagittal 3D TFE with TR/TE = 7.08/3.21 ms, FOV = 256 × 256 mm^2^ in 170 slices, each 1 mm thick; in-plane resolution = 1 × 1 mm. During the verbal fluency task, axial GE-EPI images were acquired with TR/TE = 3,000/45 ms, acquisition matrix 64 × 64 in 32 slices, each 3.75 mm thick; FOV = 240 × 240 mm^2^, resulting in 3.75 × 3.75 × 3.75 mm^3^ isometric voxels, covering all the brain.

### fMRI Analysis

The images acquired during the semantic fluency task were analyzed with the FEAT tool (MRI Expert Analysis Tool), version 6.0, from the FSL (FMRIB’s Software Library, ^1^) software. Preprocessing of the images per participant was performed by correcting for head motion with MCFLIRT (FMRIB’s motion correction linear image registration tool; Jenkinson et al., 2002). Also, non-brain tissue was removed using BET (Brain Extraction Tool; Smith, 2002). A Gaussian kernel of 6 mm FWHM for spatial smoothing and a high-pass filter of 1/60 Hz cutoff was used to remove low-frequency drift. Functional images of each participant were co-registered to structural images in native space, and structural images were normalized to Montreal Neurological Institute (MNI) standard space using the average MNI152 T1 (2 × 2 × 2 mm^3^) of FSL. The same transformation was then used to transform the functional images to MNI space.

For the first-level analysis of every participant’s performance, activations for six 24-s windows per condition were used to contrast the semantic fluency and the finger tapping conditions against the resting condition. These analyses were performed using the General Linear Model (GLM) with double-gamma hemodynamic response function (HRF), using thresholds of *Z* > 2.3 and corrected voxel significance at *P* = 0.05.

To check for issues of multicollinearity between age, sex, education, vocabulary, and MMSE, we computed the variance inflation factor (VIF) for each variable against the rest of the moderating variables. Multicollinearity diagnostics showed that all VIFs were less than 1.87 and tolerance values above 0.53, which are values within acceptable ranges (Belsley, 1991). These data suggest that multicollinearity was not a concern.

Group contrasts per condition were performed in two stages: In the first stage, (a) group and task activations were compared while controlling for age and MMSE scores as covariates of no interest. This procedure agrees with earlier studies (e.g., Hakun et al., 2015) where moderating variables showing significant group differences are entered in the analyses as nuisance covariates (see “Results” section below). In the second stage, (b) a series of analyses were conducted to evaluate the effects of sex, years of education, and vocabulary scores, with age and MMSE scores, regressed out. In this way, we assessed the interactions or effects of every single variable by group and condition. As part of this stage, we also evaluated the independent interactions of the group with age and MMSE per condition. A Different Offset/Different Slope design was used to test for interactions between the groups and the covariates per condition. For these group-covariate interactions, the FMRIB’s Local Analysis of Mixed Effects (FLAME 1 + 2) estimation method was used to create and compare group clusters (gclusters), with thresholds set at *Z* > 2.3 for the Z statistical maps and corrected cluster significance at *P* = 0.01 (Worsley, 2001; Eklund et al., 2016).

## Results

### Session 1

#### Demographics

The groups did not differ in years of education, vocabulary, or BDI scores. Though, as shown in Table 1, the MMSE scores were significantly different between groups (*p* < 0.05).

**Table 1:** Demographics^a^.

	Young group (*n* = 15)	Healthy older group (*n* = 17)	*t*-score (30)	*p*-value
Sex (F/M)	7/8	8/9		
Age (years)	26.8 (0.9)	70.6 (0.9)		
Years of education	15.8 (0.8)	13.5 (1.0)	1.8	ns
MMSE	29.6 (0.2)	28.7 (0.3)	2.6	0.02*
Vocabulary	51.9 (1.9)	48.3 (2.2)	1.2	ns
BDI	6.4 (1.1)	5.0 (0.9)	−5.3	ns

BDI, Beck Depression Inventory; MMSE, Mini-Mental State Examination; Vocabulary, scores from the Vocabulary subtest WAIS-R. *^a^Mean values and standard errors in parentheses*. ns, non-significant. *p < 0.05.

##### Neuropsychological Testing

The results for the test battery are presented in Table 2. These data showed that younger adults performed significantly better than older adults in several tests. Such differences were observed on the Digits Span tests, which measures attention and working memory and on the most demanding part of the Stroop test that assesses executive functions. Significant differences were evident in the psychomotor function evaluated with the Purdue Pegboard Test and Finger tapping task with the participants’ right hand. No group differences were observed in the results of the latter tests carried out with the left hand. Likewise, no group differences were found for Logical Memory tests.

**Table 2:** Mean, standard error, and *t*-values for cognitive and psychomotor tests.

	Young group (*n* = 15)		Healthy older group (*n* = 17)		
Test	*M*	*SE*	*M*	*SE*	*t*_(30)_
Digits Span forward	8.40	0.49	6.81	0.41	2.51*
Digits Span backward	7.07	0.42	5.63	0.46	2.32*
Digits Span total	15.47	0.82	12.53	0.61	2.92**
Stroop Word	69.13	4.67	63.24	3.89	*ns*
Stroop Color	52.00	1.12	47.47	2.05	*ns*
Stroop Word/Color	33.33	1.47	26.82	1.49	3.08**
Purdue Pegboard
Right hand	16.07	0.38	13.59	0.52	3.74***
Left hand	15.07	0.43	12.31	0.43	4.55***
Both hands	12.53	0.43	9.94	0.30	4.99***
Assembly	36.67	1.51	23.12	0.97	7.74***
Finger tapping R	238.27	9.02	208.65	8.98	2.32*
Finger tapping L	217.93	9.04	192.18	10.97	*ns*
Logical Memory I	16.60	0.97	15.94	0.85	*ns*
Logical Memory II	15.33	0.84	14.29	0.79	*ns*

*p < 0.05, **p < 0.01, ***p < 0.001. ns, non-significant.

###### Verbal Fluency Results

Table 3 shows mean scores by specific letters/categories as well as assemble mean scores for phonemic and verbal fluency tests. As observed, there were no differences between groups in any of the parameters.

**Table 3:** Mean, standard error, and *t*-values for verbal fluency tests.

	Young (*n* = 15)		Healthy older (*n* = 15)		
Test	*M*	*SE*	*M*	*SE*	*t*_(30)_
**Phonemic fluency**
F correct	14.80	0.80	14.23	1.24	*ns*
A correct	11.80	0.73	11.23	0.78	*ns*
S correct	17.13	0.68	17.82	1.10	*ns*
Mean correct FAS	14.57	0.54	14.43	0.92	*ns*
FAS errors	0.86	0.31	0.58	0.17	*ns*
FAS perseverations	0.73	0.38	1.88	0.49	*ns*
**Semantic fluency**
Animal correct	22.73	1.36	19.35	1.20	*ns*
Fruit correct	18.00	0.96	16.52	1.11	*ns*
Professions correct	16.47	0.94	16.88	1.03	*ns*
Mean 3 categories	19.06	0.79	17.51	1.00	*ns*
Mean 3 categories errors	0.06	0.07	0.29	0.14	*ns*
Mean 3 categories perseverations	1.00	0.41	0.88	0.27	*ns*

*ns, non-significant*.

### Session 2

#### fMRI Results

The activation clusters and the respective brain regions reported correspond to the maxima per cluster as reported by the FEAT tool, labeled according to the Juelich Histological Atlas (Eickhoff et al., 2007), the Harvard-Oxford Structural Atlases (Desikan et al., 2006), and the FNIRT-normalized Cerebellar Atlas in MNI152 space (Diedrichsen et al., 2009).

#### Semantic Fluency Condition

**Figure 2:**
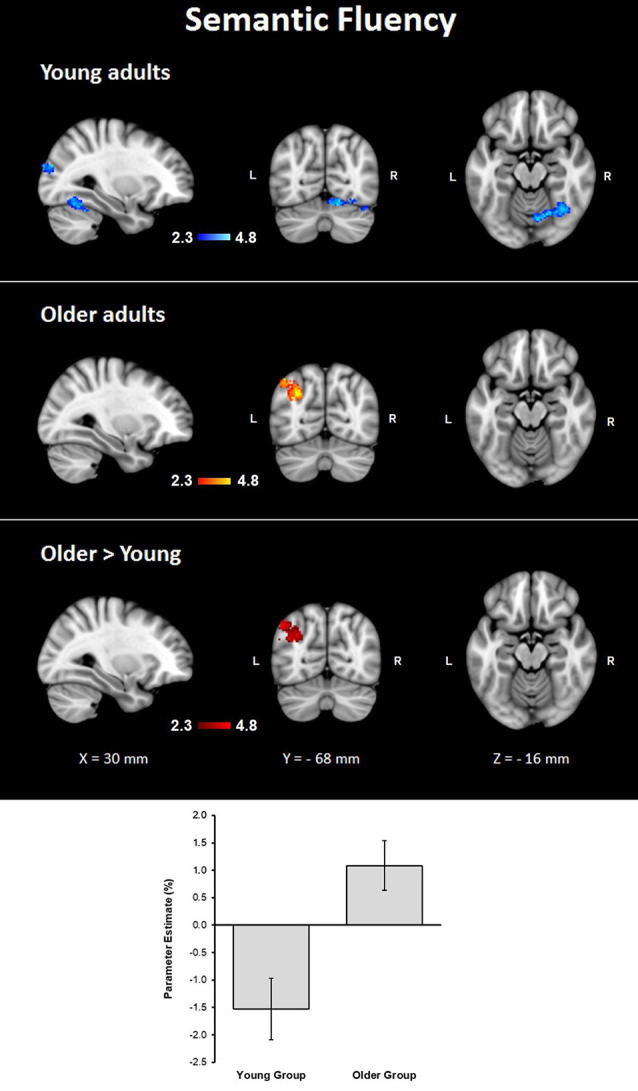
Z probability maps for group activations during the semantic fluency condition. From top to bottom: young group (blue-light blue shade), older group (red-yellow shade), and larger activations in the older group compared to the young group (red shade). Sagittal (left), coronal (center), and axial (right) neurological views are shown per contrast. Bar plots represent the mean percentage of signal change (±SEM) at the significant clusters for the corresponding comparison.

Assessment of the hemodynamic response of the young group during the verbal fluency condition contrasted against the resting condition (semantic fluency > resting) and controlled by age and MMSE scores showed activations in the right hemisphere in cerebellar crus I and VI, visual cortex (BA 17, BA 18, and V4), lateral occipital cortex, optic radiation and fornix; and bilaterally in the thalamus, anterior thalamic radiation and caudate. Meanwhile, the older group presented activations in the left inferior parietal lobule (IPL) and anterior parietal sulcus (upper side Table 4, Figure 2). When comparisons between groups were performed, larger activations were only observed in the older group with respect to the young group in the left hemisphere in the anterior intra-parietal sulcus (IPS), IPL, and lateral occipital cortex (upper side Table 4, Figure 2).

**Table 4:** Clusters for brain activations by the group during semantic fluency and finger tapping while controlling for age and MMSE.

Contrast	Voxels	*P*	Z-MAX	Z-MAX	Z-MAX	Z-MAX	Structures
			*X* (mm)	*Y* (mm)	*Z* (mm)		
***Semantic Fluency***
Young group activations	571	0.0061	4.74	42	−62	−24	R cerebellar crus I and VI, visual cx V4
	544	0.00814	4.48	10	−94	4	R visual cx BA 17 and BA 18, optic radiation, lateral occipital cx.
	418	0.0332	4.87	−10	−16	16	LR thalamus, anterior thalamic radiation, caudate; R fornix.
Older group activation	618	0.00373	4.29	−44	−68	50	L inferior parietal lobule and intra-parietal sulcus.
*O > Y*	838	0.0004	4.72	−44	−68	50	L anterior intra-parietal sulcus, inferior parietal lobule, lateral occipital cx.
**Finger Tapping**
Young group activations	1,498	0.00000131	8.25	−14	−50	−12	L cerebellar V and VI, lingual gyrus, visual cx V4; LR cerebellar I–IV.
	711	0.00127	4.81	12	−90	10	R visual cx BA sulcus; and bilaterally, in premotor cortex17 and BA 18, optic radiation.
Older group activation	401	0.037	4.6	−56	−20	46	L primary somatosensory cortex, premotor cx, primary motor cx, inferior parietal lobule, anterior intra-parietal sulcus.
*Y > O*	1,115	3.01 e-5	4.52	−14	−50	−12	L cerebellar V, lingual gyrus, cingulate gyrus, hippocampus, callosal body, and fornix.

*Y, young group; O, older group. All the comparisons were performed controlling for MMSE score and age*.

#### Finger Tapping Condition

**Figure 3:**
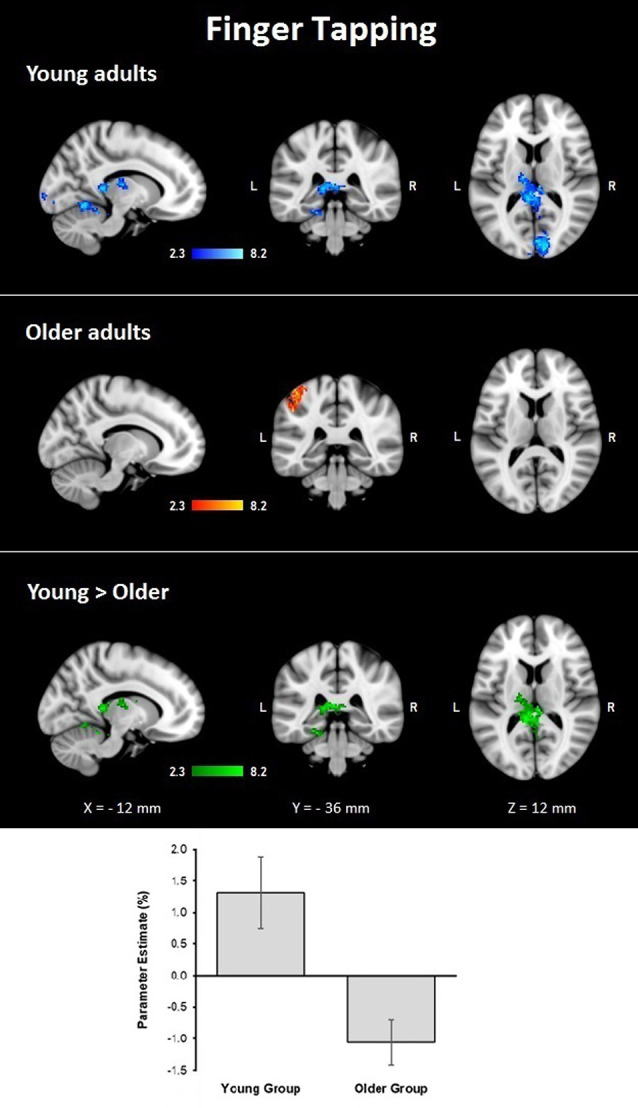
Z probability maps for group activations during the finger-tapping condition. From top to bottom: young group (blue-light blue shade), older group (red-yellow shade), and larger activations in the young group compared to the older group (green shade). Sagittal (left), coronal (center), and axial (right) neurological views are shown per contrast. Bar plots represent the mean percentage of signal change (±SEM) at the significant clusters for the corresponding comparison.

During the finger tapping condition, the young group showed activations in right visual cortex BA 17 and BA 18 and optic radiation; left cerebellar V and VI, lingual gyrus, and visual cortex V4; and bilateral cerebellar I–IV, when controlling for age and MMSE scores. The older group showed activations after controlling for age and MMSE scores in the left hemisphere in the primary somatosensory cortex, premotor cortex, primary motor cortex, IPL, and anterior IPS (lower side Table 4, Figure 3). The young group presented larger activations in left cerebellar V, lingual gyrus, cingulate gyrus, hippocampus, callosal body, and fornix (lower side Table 4, Figure 3). Comparisons between groups controlling for the covariates showed no larger activations in the older group compared to the young group.

#### Comparisons Between Tasks

**Figure 4:**
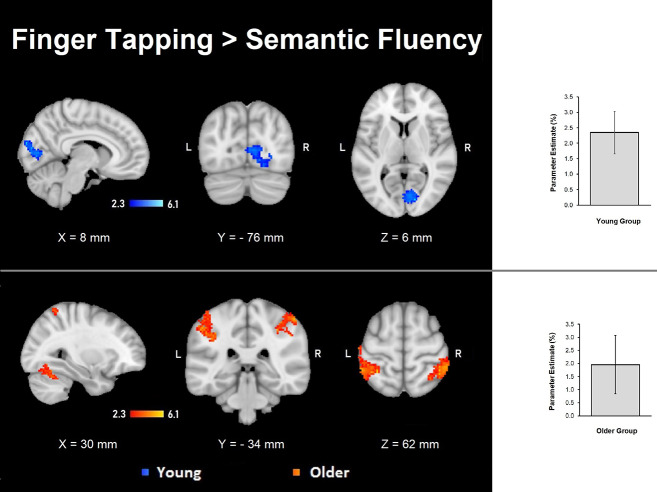
Z probability maps of the significant contrasts comparing the semantic fluency and finger tapping conditions. No significant differences between groups were found either for semantic fluency > finger tapping or finger tapping > semantic fluency. The finger tapping > semantic fluency contrasts showed only separate significant activations for the young group (top illustration, blue-light blue shade) and the older group (bottom illustration, red-yellow shade). Sagittal (left), coronal (center), and axial (right) neurological views per contrast are shown. Bar plots, in the right, represent the corresponding mean percentage of signal change (± SEM) at the significant clusters. The significant cluster for the young group showed *p* < 0.029 (mean parameter estimate, PE = 2.345; mean *z* = 2.943), while the same cluster in the older group showed no significant values (PE = − 1.12; mean *z* = − 1.643). Conversely, the three significant clusters in the older group were significant (PE = 1.955; *p* < 0.029; mean *z* = 2.942) while corresponding values for the younger group were not (PE = − 1.343; mean *z* = − 1.796).

The semantic fluency condition did not show any significantly increased activation compared to the finger-tapping condition either per group or between groups. Finger tapping compared to semantic fluency showed significantly increased activation in the young group in a cluster in the right visual cortex. In contrast, the older group showed three significant clusters of increased activation during finger tapping compared to semantic fluency in right cerebellum (V and VI) and temporal occipital fusiform cortex, and bilateral anterior IPS, IPL, primary somatosensory cortex and SPL (Table 5, Figure 4). However, no significant differences between groups in the finger tapping > semantic fluency contrast were found.

**Table 5:** Clusters for significant differences between finger tapping and semantic fluency.

Contrast	Voxels	*P*	Z-MAX	Z-MAX	Z-MAX	Z-MAX	Structures
			*X* (mm)	*Y* (mm)	*Z* (mm)		
**Finger Tapping > Semantic Fluency**
Young group	438	0.029	4.47	14	−82	6	R visual cx BA 17and BA 18
	860	0.0004	4.96	−44	−36	42	L anterior intra-parietal sulcus, inferior parietal lobule, primary somatosensory cx, superior parietal lobule
Older group	601	0.005	4.74	48	−38	58	R inferior parietal lobule, primary somatosensory cx, superior parietal lobule, anterior intraparietal sulcus
	438	0.029	4.35	16	−52	−20	R cerebellar V and VI, temporal occipital fusiform cx

*Comparisons were performed controlling for MMSE score and age*.

#### Effects and Interactions of Sex, Education, and Vocabulary in Semantic Fluency and Finger Tapping

In the second step of the analyses, the effects of sex, years of education, and vocabulary scores were evaluated by regressing each of them in the model while controlling for age and MMSE scores.

##### Semantic Fluency

**Figure 5:**
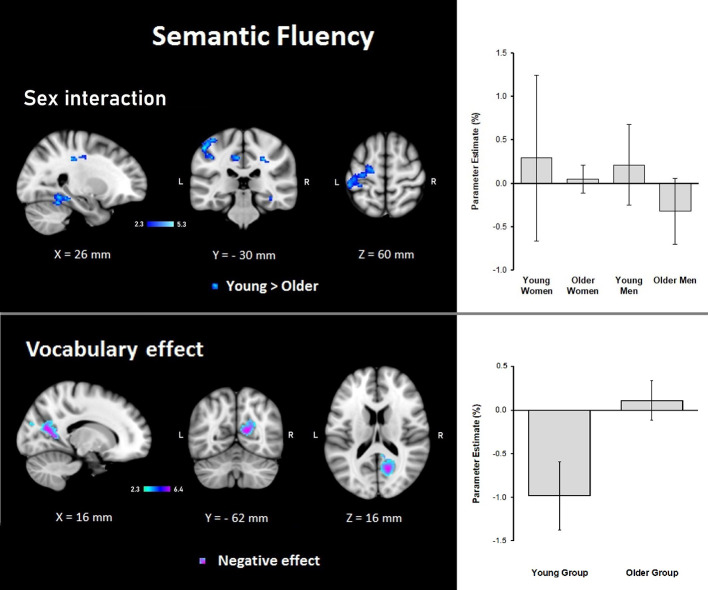
Z probability maps for steeper group × sex interactions in the young group (top), and the vocabulary global negative effect (bottom) during the semantic fluency condition. Sagittal (left), coronal (center), and axial (right) neurological views are shown per contrast. Bar plots represent the mean percentage of signal change (±SEM) at the significant clusters for the corresponding comparison.

Results demonstrated that there were interactions between group and sex and the general effects of vocabulary scores on the hemodynamic response. For sex, women showed a larger effect than men (data not shown). However, interactions between group and sex were found, showing steeper slopes for the young group, at three clusters in the right hemisphere in the temporal occipital fusiform cortex, lingual gyrus, parahippocampal gyrus, hippocampus, cingulate gyrus, SPL, cerebellar V and VI, optic radiation and superior longitudinal fascicle; in the left hemisphere in the primary somatosensory cortex, IPL, and anterior intraparietal sulcus; and bilaterally, in premotor cortex (BA 6), the primary motor cortex (BA 4), and corticospinal tract (Table 6, Figure 5). No significant effects or interactions on the BOLD signal for semantic fluency were found while testing for years of education. However, for vocabulary, a negative effect on activations during the semantic fluency condition was found in the right hemisphere in the visual cortex (BA 17 and BA 18), cingulum, optic radiation, and callosal body (Table 7, Figure 5).

**Table 6:** Sex interactions in semantic fluency and finger tapping.

Sex interactions	Voxels	*P*-value	*Z*-Values peak	MNI coordinates			Structures
				*X*	*Y*	*Z*	
**Semantic Fluency**
Y > O	618	0.00306	3.77	−50	−30	52	L primary somatosensory cx, inferior parietal lobule, premotor cx BA 6, primary motor cortex BA 4, anterior intraparietal sulcus, corticospinal tract.
	468	0.016	5.00	30	−48	−20	R temporal occipital fusiform cx, lingual gyrus, parahippocampal gyrus, cerebellar V and VI, hippocampus, optic radiation.
	437	0.023	3.69	4	−20	42	R cingulate gyrus, premotor cx BA 6, primary motor cx BA 4, superior parietal lobule, corticospinal tract, superior longitudinal fascicle.
**Finger Tapping**
Y > O	948	0.0001	4.83	46	−82	30	R inferior parietal lobule, anterior intra-parietal sulcus.
	492	0.0115	3.67	−38	−76	36	L inferior parietal lobule, anterior intra-parietal sulcus, superior parietal sulcus.
O > Y	589	0.003	3.97	28	−90	38	R lateral occipital cx, visual cx BA 18.

*Y, young group; O, older group; Y > O, steeper interaction slope for the young group; O > Y, steeper interaction slope for the older adult group. All the comparisons were performed controlling for MMSE score and age*.

**Table 7:** Effects and interactions of vocabulary and years of education in semantic fluency and finger tapping.

	Voxels	*P*-value	*Z*-Values peak	MNI coordinates			Structures
				*X*	*Y*	*Z*	
**Vocabulary**
Semantic Fluency
The negative effect for both groups	589	0.00495	3.88	10	−48	6	R visual cx BA 17 and BA 18, cingulum, optic radiation, callosal body.
Finger Tapping
Interaction O > Y	555	0.00577	4.02	−46	24	36	L Broca’s area BA 44 and BA 45, middle frontal gyrus, superior frontal gyrus.
	515	0.00901	4.18	24	20	56	R superior frontal gyrus, middle frontal gyrus, premotor cx BA 6.
	477	0.0139	3.96	−42	−84	30	L inferior parietal lobule, lateral occipital cx.
**Years of Education**
Semantic Fluency
-	-	ns	ns	-	-		
Finger Tapping
Interaction O > Y	402	0.0355	3.96	14	−72	34	R cuneal cx, L cingulate gyrus, callosal body, RL precuneus, superior parietal lobule.

*Y, young group; O, older group; Y > O, steeper interaction slope for the young group; O > Y, steeper interaction slope for the older adult group. All the comparisons were performed controlling for MMSE score and age*.

##### Finger Tapping

**Figure 6:**
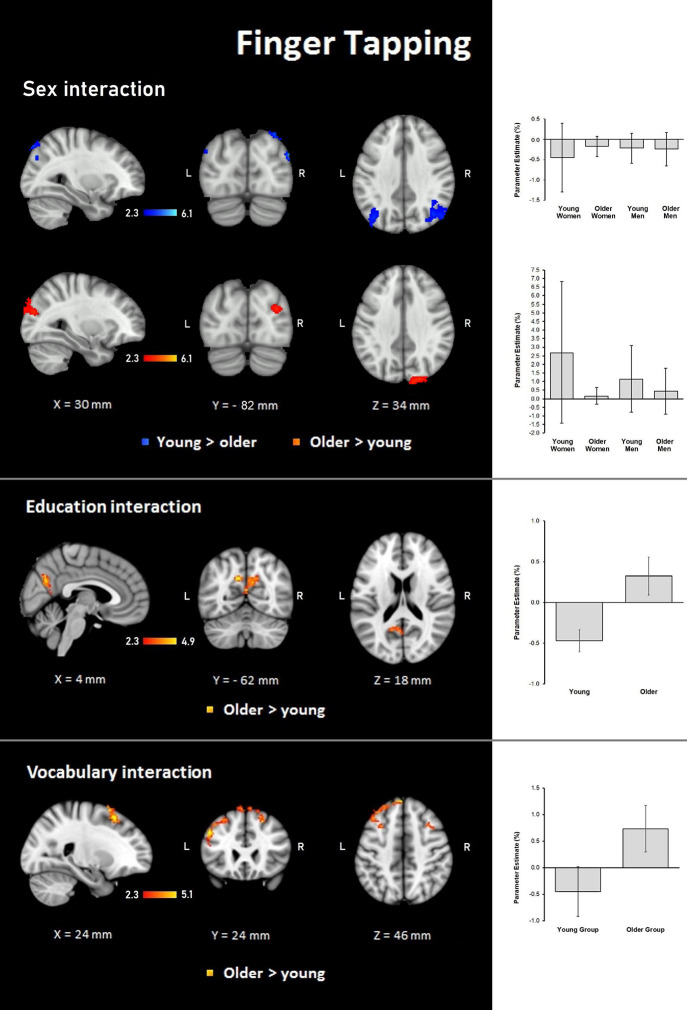
Z probability maps for steeper group × sex interactions in the young group (top), steeper group × sex interactions in the older group (second row), steeper group × education interactions in the older group (third row), and steeper group × vocabulary interactions in the older group (bottom) during the finger-tapping condition. Sagittal (left), coronal (center), and axial (right) neurological views are shown per contrast. Bar plots represent the mean percentage of signal change (±SEM) at the significant clusters for the corresponding comparisons.

Results demonstrated significant interactions between group and all three evaluated variables on the hemodynamic response during the finger-tapping condition. For sex, a steeper interaction slope for the young group as compared to the older group was found in two clusters in bilateral IPL and anterior IPS and left SPL. The older group showed steeper interaction slopes compared to the young group in the right lateral occipital cortex and visual cortex (BA18; Table 6 and Figure 6).

For education, the interaction slope was only sharper for the older compared to the young group in the right cuneal cortex, left cingulate gyrus and callosal body, and bilateral precuneus cortex and SPL (Table 7 and Figure 6).

The vocabulary interaction slope was steeper only for the older group compared with the young group in three clusters in the right hemisphere in premotor cortex (BA 6); in the left hemisphere in Broca’s area (BA 44 and BA 45) and IPL, and lateral occipital cortex; and bilaterally in middle and superior frontal gyri (Table 7 and Figure 6).

#### Age and MMSE Interactions

Age and MMSE showed interactions with the group for both the semantic fluency and finger tapping conditions; however, there was a larger number of significant voxels during the semantic fluency condition.

##### Age

**Figure 7:**
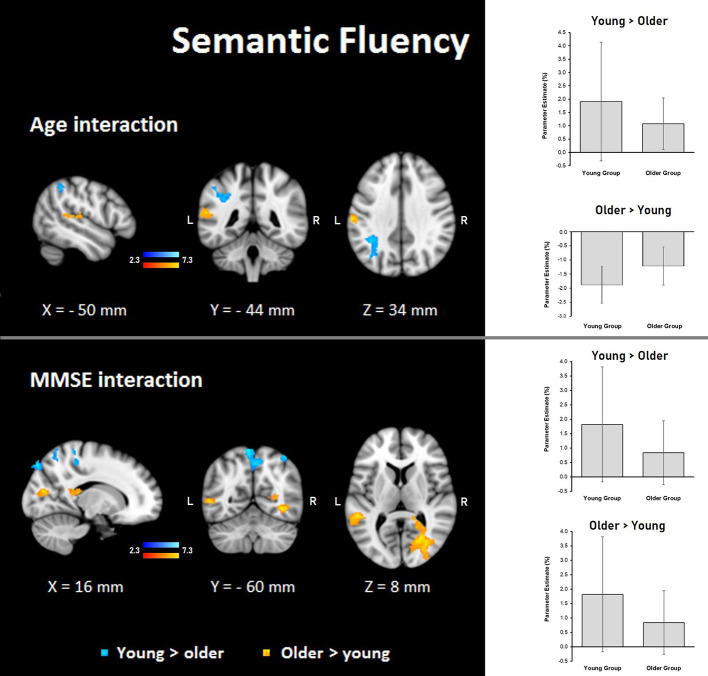
Z probability maps for interactions of the group with age (top) and MMSE score (bottom) during the semantic fluency condition. Steeper slopes of the group × age/group × MMSE score interactions for the young group (in a blue-light blue shade), and steeper slopes of the group × age/group × MMSE score interactions for the older group (in a red-yellow shade). Sagittal (left), coronal (center), and axial (right) neurological views are shown per contrast. Bar plots represent the mean percentage of signal change (±SEM) at the significant clusters for the corresponding comparisons.

Semantic fluency: during the semantic condition, steeper interaction slopes between group and age for the young were found in the left IPS, anterior IPS, SPL, and lateral occipital cortex. On the other hand, steeper interaction slopes between group and age for the older adults were found in the left secondary somatosensory cortex, IPL, primary somatosensory cortex, and primary auditory cortex (Table 8, Figure 7).

**Table 8:** Clusters for control covariate, age, and MMSE, interactions.

	Voxels	*P*-value	*Z*-Values peak	MNI coordinates			Structures
				*X*	*Y*	*Z*	
**Age**
Semantic fluency
Y > O	772	0.000752	7.36	−46	−48	44	L Inferior parietal sulcus, anterior intra-parietal sulcus, superior parietal lobule, lateral occipital cx.
O > Y	468	0.0179	5.84	−64	−24	28	L Secondary somatosensory cx/parietal operculum, inferior parietal lobule, primary somatosensory cx BA 1 and 2, primary auditory cx.
Finger-tapping
Y > O	403	0.0352	4.22	12	−20	70	R Premotor cx BA 6, corticospinal tract, primary motor cx BA 4, primary somatosensory cx BA 3.
**MMSE**
Semantic fluency
Y > O	1718	2.98 e-7	5.10	−2	−52	66	LR Superior parietal lobule, primary somatosensory cx, primary motor cx; R occipital pole, lateral occipital cx.
O > Y	1592	7.15e-07	5.81	40	−58	−6	R Optic radiation, lateral occipital cx, temporal occipital fusiform cx, inferior temporal gyrus, callosal body.
	538	0.00824	6.09	−48	−54	−2	L Superior longitudinal fasciculus, middle temporal gyrus.
Finger-tapping
O > Y	522	0.00892	3.69	46	28	22	R Middle frontal gyrus, Broca’s area BA 44 and BA 45, inferior frontal gyrus, superior longitudinal fasciculus, premotor cx BA6.
	374	0.05	4.24	30	−80	2	R Lateral occipital cx, optic radiation, callosal body, inferior longitudinal fasciculus, inferior fronto-occipital fasciculus.

*Y, young group; O, older group; Y > O, steeper interaction slope for the young group; O > Y, steeper interaction slope for the older adult group*.

**Figure 8:**
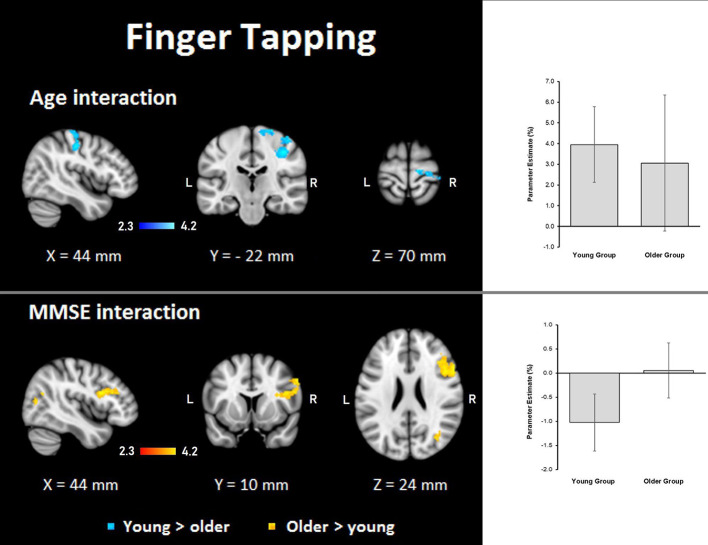
Z probability maps for interactions of the group with age (top) and MMSE score (bottom) during the finger-tapping task. In the top, steeper slopes of the group × age interaction for the young group (in a blue-light blue shade). In the bottom, steeper slopes of the group × MMSE score interaction for the older group. Sagittal (left), coronal (center), and axial (right) neurological views are shown per contrast. Bar plots represent the mean percentage of signal change (±SEM) at the significant clusters for the corresponding comparisons.

Finger tapping: age showed steeper interaction slopes with the group only for the young, which were located in the right premotor cortex (BA 6), corticospinal tract, the primary motor cortex (BA 4), and primary somatosensory cortex (BA 3; Table 8, Figure 8).

##### MMSE

Semantic fluency: the MMSE scores showed steeper association slopes for the young during the semantic fluency task in bilateral SPLs, primary somatosensory, and primary motor cortices, and the right occipital pole and lateral occipital cortex. Also, steeper group × MMSE interaction slopes for the older adults in the semantic fluency task were found in two clusters in the right hemisphere in optic radiation, lateral occipital cortex, temporal occipital fusiform cortex, inferior temporal gyrus, and callosal body, as well as in the left hemisphere in superior longitudinal fasciculus and middle temporal gyrus (Table 8, Figure 7).

Finger tapping: MMSE scores showed only steeper interaction slopes with the group for older participants; these steeper interaction slopes were present in two clusters in the right middle frontal gyrus, Broca’s area (BA 44 and BA 45), inferior frontal gyrus, superior longitudinal fasciculus, premotor cortex (BA 6), lateral occipital cortex, optic radiation, callosal body, inferior longitudinal fasciculus, and inferior fronto-occipital fasciculus (Table 8, Figure 8).

## Discussion

We examined the role of different demographic variables as well as proxies of cognitive reserve on the hemodynamic brain response during the execution of semantic fluency as compared to a simple motor task i.e., finger tapping. The overall finding shows that the role of demographics and proxies of CR on the performance of both tasks is intricate and not entirely opposed as one might expect from two different tasks. Due to the nature of the results, we will first discuss the findings of brain activation when controlling for age and MMSE, then we will address the effects of each demographic/CR proxy by type of task.

### Semantic Fluency and Finger Tapping While Controlling for Age and MMSE

Behavioral results showed that the group of active older adults had no difficulties in generating words based on a particular category or a particular letter. No significant group differences were observed in any of the parameters of verbal fluency, in spite that older adults had lower scores than younger participants on executive functions and fine motor control. The imaging data did show group differences in brain activation during covert semantic fluency when controlling for age and MMSE. In the younger group, increased activation of subcortical areas including bilateral thalamus and the caudate as well as the occipital cortex and visual areas were observed, which agrees with earlier reports (Hwang et al., 2009; Li et al., 2017). Cerebellar areas were also activated, which has been reported during covert and overt production of semantic verbal fluency (Gurd et al., 2002; Nagels et al., 2012). Specifically, the right cerebellum was involved during verbal fluency performance, which is in agreement with previous research (Starowicz-Filip et al., 2017).

In the older group, we found that there were fewer areas recruited during semantic fluency as compared to the younger group; however, there were also additional activations. These differences in activation patterns have been reported recurrently in the past (e.g., Baciu et al., 2016). Nevertheless, in our study, the main finding was the appearance of similar activation patterns of the left anterior IPS and left IPL in both verbal fluency and finger tapping. The IPS is an area associated with the planning of grasping movements while the IPL is involved in the integration of sensorimotor processes (Kaas, 2012). Nonetheless, in humans, both areas on the left hemisphere subserve the integration of language and sensorimotor information (Hier et al., 1987). Specifically, the IPS has been related to semantic selection (Whitney et al., 2012) and interestingly, the increased activation found on the IPS among older adults during semantic verbal fluency has been reported earlier (Destrieux et al., 2012).

These data suggest that our design facilitated the activation of IPS and IPL due to a reciprocal influence of the tasks. In the older group, this influence was strong enough to evoke recruitment of areas subserving both motor and language processes, while the younger group in a higher degree preserved separate patterns of brain activations even though the younger participants presented continuous activations in visual cortex and cerebellum across conditions. We interpret the lack of specificity in brain functioning among older adults as a result of dedifferentiation mechanisms occurring in aging, which may compensate for neural deterioration (Li, 2002).

The behavioral findings from the finger tapping showed that older adults were slower on tapping with their right finger, while they displayed significant dexterity declines on the Purdue Pegboard task. These findings are mostly in agreement with the literature (Serbruyns et al., 2015). Still, since the older adults in our study were very active persons, they may have preserved good control of the non-dominant hand for simple repetitive movements which explain the lack of age-differences on left finger tapping.

As for the imaging data, we again observed different patterns of activation between groups. We observed higher activation in subcortical regions among younger participants, which contrasts with earlier studies reporting few age-differences during simple finger tapping (e.g., Wu and Hallett, 2005). However, it has been reported that age differences on finger tapping rely on the type of task applied. A meta-analysis on functional imaging and finger tapping showed that the type of tapping task matters when determining its underlying brain network (Witt et al., 2008). In the present study, we employed a simple bimanual finger-tapping task that was paced by visual cues. Areas reported to be activated under these circumstances include occipital areas, ventral premotor cortex, the posterior cerebellum, and vermal lobes (Witt et al., 2008). Also, the supplementary motor cortex and basal ganglia are activated, unrelated to the type of task. Results from our younger group, therefore, corroborated with the activations mentioned above. However, younger participants had higher activation in the visual cortex. Finally, additional activation on subcortical regions in the younger group including the cingulum and hippocampus agrees with previous data reporting activation of these regions during learning of motor sequences and finger tapping (Albouy et al., 2012). As an ensemble, the behavioral and imaging results suggest that finger tapping is not a simple task for older adults. Their performance in laboratory conditions revealed declined finger tapping capacity while their hemodynamic brain activation during finger tapping was lower than in younger participants. It is important to remind that finger tapping assessed outside the scanner was a unimanual task, while finger tapping assessed during scanning was a bimanual task. Still, these were unexpected findings that cannot be easily explained. Even though we did not score finger tapping performance during brain scanning, we verified that all participants performed the bimanual finger tapping during scanning time, which points to a reduced brain activation without evident fault in performance. Our interpretation is that older adults demonstrated lower BOLD activation due to reduced brain resources resulting from normal brain deteriorations (Cabeza et al., 2018). Despite lower activation, older participants performed the task without difficulty. Whether their performance during the scanner was qualitatively different from that of younger participants (e.g., slower number of taps or less coordination) we cannot evaluate this, and slighter but clear differences in this task may be related to the decreased activation. Future studies may carry out an advanced analysis on this matter. Taken together, the behavioral and fMRI results suggest declined finger tapping ability in older adults (Reuter-Lorenz and Cappell, 2008).

### Comparisons Between Semantic Fluency and Finger Tapping

Contrasting our tasks yielded significant results by group only when finger tapping was compared with semantic fluency. The reverse contrast did not yield any significant result. Thus, the significant findings demonstrated greater neural resources recruited during the finger-tapping task, which generated age-specific patterns of activations. These age-specific activations were more widespread in older adults than in younger participants. A possible explanation for this outcome is that the explicit motor component demanded during finger tapping exert higher demands in older individuals than mental recall. In the past, it has been demonstrated that older adults recruit larger brain areas during simple motor tasks (Mattay et al., 2002). Also, findings from fMRI studies evaluating mental motor imagery vs. actual motor execution of the same tasks support the hypothesis that the actual execution of finger tapping requires more cognitive resources in older individuals. For example, Zapparoli et al. (2013) demonstrated the existence of larger brain networks activated during motor execution of finger movements than mentally evoked finger movements in healthy older adults. This was not the case for younger individuals. During the actual motor execution of this task, various areas were activated in both younger and older participants, such as the premotor cortex, supplementary motor area (SMA), parieto-temporal operculum, and anterior parietal cortex. However, in older adults, additional activations occurred in left occipital gyrus, paracentral lobule, and premotor cortex. Also, greater activation in the older group was found in SMA and right cerebellum.

Although our study differs in many ways from the study of Zapparoli et al. (2013), we regard our findings as broadly replicating their results since we demonstrated an extended brain network activated only in the older adults during overt execution of finger tapping. This similarity occurred even though we had a verbal fluency task as the contrasting “mental task.” These authors also suggested that motor execution of their finger movement task imposed higher cognitive demands in older persons than in young, which is in line with our idea that finger tapping is not a simple task for older adults. Finally, the fact that the cerebellum, IPS, and IPL were the areas significantly activated in the older group during finger tapping when contrasted against semantic fluency, only emphasizes the important role of these specific brain structures in the paradigm employed in the present study.

### The Effects of Sex, Vocabulary, and Education

Results demonstrated that sex is a relevant demographic variable for brain activation in both verbal fluency and finger tapping among younger participants. Stronger activations were observed in both hemispheres in the temporoparietal and occipital regions of younger females. An interesting distinction due to the nature of the task regards the activation of the right fusiform cortex, parahippocampal gyrus, and hippocampus during semantic verbal fluency, which according to the literature, are areas of visual word-form recognition and working memory (Caspers et al., 2015). Possibly, younger participants recreate images of the semantic category presented on the screen, which would be comparable to object identification or categorization. These abilities are consistently related to activation of the mentioned brain regions, especially the fusiform cortex (Aminoff et al., 2013). Alternatively, some of these areas responded to task switching (Kim et al., 2012).

Another issue of interest in younger adults is the overlapping activation of left IPL and IPS in both tasks. As described in the previous section, these two areas showed activations in the older group when sex was not entered in the model. Thus, when sex is used as a predictor, we observed an association with IPL, IPS, somatosensory cortices, cerebellum, and occipital regions again in the younger group. These findings would indicate that younger males were the ones lacking activations in IPL and IPS across conditions. Younger males have been reported to be better than younger women in various visual tasks and this could explain to some degree the present results, but since sex differences in visual abilities are still unresolved (Shaqiri et al., 2018) future studies should verify the issue. Similarly, we found an additional sex effect in older adults showing additional activation of occipital and visual cortices during finger tapping in females. Whether this indicates that older females are more sensitive to visual stimuli or rely more on visual areas to accomplish the tasks also needs to be confirmed in forthcoming research.

Regarding the effects of education, we found no association in any of the groups between this variable and semantic verbal fluency. However, vocabulary showed a negative correlation in both groups for semantic verbal fluency. This association showed that lower vocabulary was coupled with a higher BOLD response in occipital regions. These results corroborated findings reported by Nagels et al. (2012) who reported a lack of influence of education on semantic verbal fluency but a significant negative association for brain activation and vocabulary. Nevertheless, the areas of activation differed between our data and the mentioned study possibly due to sample type as they tested only healthy younger adults, while we included an older group. Also, these authors employed an overt semantic fluency task and not a covert task. In our study, the lack of significant relationships between education and semantic verbal fluency should be understood as the result of including well-educated and active seniors in the study. Still, the important finding is that vocabulary turned out to be the relevant proxy of CR for brain activation during semantic fluency. Because visual cortices and occipital regions were activated due to the visual cues signalizing type of tasks, the effects of vocabulary on the BOLD response of semantic verbal fluency suggest that the higher the vocabulary is, the less activated become the visual regions. Accordingly, brain activation in participants with the higher vocabulary of any age group was low, indicating that these participants coped better with task demands by allocating less neural activation in occipital/visual areas during semantic word generation. This is in line with the suggestion that individuals with low lexical abilities draw upon compensatory processes, such as visual imagery (Swanson and Trahan, 1996).

Interestingly, the effects of education and vocabulary existed in the older group during the finger-tapping task in which higher education was associated with higher activation in parietal areas including cingulate gyrus and precuneus. Activation of these areas agrees with pioneer imaging studies in aging demonstrating that increasing age associates with increased activation in the precuneus and cingulate gyri (Grady et al., 2006), especially during the execution of working memory tasks (Archer et al., 2018). Hence, it could be argued that our design posed working memory demands to older participants due to the shifting between verbal fluency and finger tapping in relatively short periods. Based on the “Compensation-Related Utilization of Neural Circuits Hypothesis” (CRUNCH; Reuter-Lorenz and Cappell, 2008), we believe that increased brain activity occurred to meet each task’s requirements. As already stated, we did not measure the accuracy of finger tapping during the scanning session. However, we ensured that all participants appropriately executed the task, and thus, the higher activation reflects a compensatory mechanism (Archer et al., 2018).

In line with these findings, a positive association also existed between vocabulary and higher neural activations in the older group during finger tapping in Broca’s area, middle and superior frontal gyri, premotor cortex, and parieto-occipital regions. It is worth noting that language and frontal areas were activated during the motor task, which could denote dedifferentiation mechanisms taking place in older people to adopt optimal performance, and in this case, during finger tapping. Though, other interpretations exist. For instance, activation of language areas could be related to a carry-over effect specific to the older group due to changes in the deployment of the BOLD response (West et al., 2019). Alternatively, it is possible that older participants remained unconsciously thinking about semantic categories during the performance of the motor task or that they covertly pace themselves using inner speech/sub articulation.

### The Effects of Age

As expected, “age” used as a predictor of brain activation showed different outcomes than those reported when it was used as a nuisance variable. For the semantic verbal fluency, the main difference was the evident associations within the parietal regions that differ by group. Younger adults displayed stronger associations with inferior parietal sulcus, anterior intra-parietal sulci, and SPL, while the older group had stronger associations in IPL and operculum. All these areas have been related to a distributed language network underlying language processes, such as speech comprehension (Price, 2012), language production (Geranmayeh et al., 2012) as well as phonological and semantic processing (Fuertinger et al., 2015). Notwithstanding, the relationship between older age and IPL is notable. As mentioned previously, IPL is related to sensorimotor integration, but it is also involved in language functions, such as bilingualism and polyglot abilities (Abutalebi et al., 2015). Loss of gray matter in IPL has been linked to mild cognitive impairment and conversion to dementia (Jacobs et al., 2012). In turn, increments of gray matter due to bilingualism in IPL are suggested to be neuroprotective in healthy older persons (Abutalebi et al., 2015). Thus, our sample of older adults deployed a strong relationship between activations in IPL and semantic verbal fluency and even though we did not calculate cortical volumes, possibly our older participants had a normal age-related loss of gray matter in IPL as reported in the literature (Taki et al., 2011). In such a case, the higher neural response in this context will denote a compensatory mechanism to achieve semantic retrieval (Grady, 2012) since they showed comparable verbal fluency abilities than younger participants outside the scanner.

Data revealed that during semantic verbal fluency, several somatosensory areas were also correlated with age, specifically in the older group. Because somatosensory processes are acknowledged as important in the control of hand movements (Gardner et al., 2007), possibly a continuous activation of these areas existed in older participants to cope with the interchangeable performance of semantic verbal fluency and finger tapping. Finally, and not unsurprisingly, the effects of age during finger tapping existed for the older group related to the activation of motor regions. These findings agree with the idea of increased brain activation as a compensatory process.

### The Effects of MMSE

Global cognitive status as measured with MMSE scores has shown to predict semantic verbal fluency (Obeso et al., 2012). Since semantic verbal fluency is recurrently included in batteries for the detection of dementia, and MMSE is the golden instrument for the detection of pathological cognitive decline, we decided to explore how MMSE correlated with the hemodynamic brain response of verbal fluency and finger tapping. Results demonstrated differences in patterns of association between groups. In the younger group, MMSE was positively associated with activation in occipital regions, somatosensory and motor cortices, and notably with SPL during semantic verbal fluency. This latter structure was also relevant for the young group in the finger-tapping test. Activation on SPL is related to attention, working memory, and visual perception (Wang et al., 2015). As anticipated, younger participants had almost no variation in MMSE scores. Most young participants scored 30, even if exceptions existed. Thus, higher MMSE scores were related to higher activation on SPL and therefore, better attentional control.

As for the older group, they displayed similar associations across verbal fluency and finger tapping between MMSE regarding brain activation in visual regions, which was related to stimuli presented in the scanner. However, the MMSE scores of the older group presented more variations than the younger group. Higher scores were associated with areas of middle temporal gyrus, which is an important area for semantic generation, naming (Indefrey and Levelt, 2004) and in general for semantic tasks (Price, 2010). In finger tapping, higher MMSE scores were related to stronger activation on Broca’s area and premotor regions, which suggest an influence of semantic verbal fluency on the motor task. All in all, these data corroborate the relevance of global mental cognition on semantic verbal fluency across groups and on finger tapping in the older group. Accordingly, these data reinforce the idea that older adults need to recruit several regions to perform tasks of a varied nature.

### Limitations of the Study

We need to acknowledge the limitations inherent to the covert speech paradigm adopted in our study. As in any covert paradigm, the main shortcoming is the impossibility to register the actual responses of the participants. Aside from the evident problem of not having behavioral data, some authors claim that there is an uncertainty of whether the neural activations observed are uniquely related to the tasks in question (Gracco et al., 2005). In our data, many of the observed brain activations indeed overlap with processes related to the reading of words and visual processing of the stimuli. However, these processes are part of the experimental situation and by requiring inner speech production we avoid unnecessary head/orofacial movements related to word articulation. Future studies should explore the effects of moderating variables on an overt speech paradigm as a complementary approach to the present findings.

## Conclusion

The present results demonstrated the important role of parietal regions, especially the IPL during the execution of a covert semantic verbal fluency task and a finger-tapping task. Its role is masked in younger individuals when controlling for variables where age differences exist. It was evident that central demographic variables on the BOLD response during semantic verbal fluency were age and sex, while the only proxy of CR showing a significant association across age groups was vocabulary. Thus, vocabulary and not education should be considered when analyzing hemodynamic brain activations related to semantic word generation. Finally, our data raised the question about how demographics and variables of CR modulate brain activity of a simple motor task. The findings suggest that all the moderating variables accounted for in our study were relevant for the finger tapping brain response.

The effects of moderators will shape the outcome of a study, and at present, there are no standard approaches to deal with these issues. In most of the studies, the automatic correction for aging and/or sex is performed as customary, even if there is no substantial evidence for conducting these corrections. The approach of “adjusting variables of no interest” in aging studies should not be applied without *a priori* evidence justifying such statistical maneuvers. The present study is good evidence that due to behavioral and neural dedifferentiation occurring in aging, the moderating effects of demographics and CR proxies are complex and differ from one task to another. Therefore, the role of demographics and other moderating variables should be evaluated in the functional imaging of older adults.

## Data Availability Statement

The datasets for this article cannot be made publicly available due to restrictions of the Norwegian Research Ethics Committee. However, derived data such as raw statistical maps can be obtained from C. Rodríguez-Aranda, claudia.rodriguez-aranda@uit.no.

## Ethics Statement

The studies involving human participants were reviewed and approved by the local Research Ethics Committee of North-Norway (REK-Nord). The patients/participants provided their written informed consent to participate in this study.

## Author Contributions

CR-A is the PI of this research. CR-A and TV contributed with the conception and design of the study. CR-A, KW, RE, and TV collected the data and organized the database. CR-A, SC-C, and TV performed the statistical analysis. FB and TV performed critical revision of the methods. CR-A and SC-C wrote the first draft of the manuscript. TV and FB wrote sections of the manuscript. FB and KW revised intellectual content. All authors contributed to manuscript revision, read, and approved the submitted version.

## Conflict of Interest

The authors declare that the research was conducted in the absence of any commercial or financial relationships that could be construed as a potential conflict of interest.

## Footnotes

^1^www.fmrib.ox.ac.uk/fsl
