# Supplementary material for: The Role of Moderating Variables on BOLD fMRI Response During Semantic Verbal Fluency and Finger Tapping in Active and Educated Healthy Seniors
Source: Front Hum Neurosci. 2020 Jun 5;14:203. doi: 10.3389/fnhum.2020.00203 (PMC7290010; doi:10.3389/fnhum.2020.00203)
Supplement: Supplementary file 1 [file Table_1.DOCX]

Supplementary Material

# Supplementary Table 1

| Table 1. Demographics and semantic fluency scores for pilot data on three additional semantic categories. ^a^ | | | | |
| --- | --- | --- | --- | --- |
|  | **Young group**  **(n = 22)** | **Healthy older group**  **(n = 15)** | **t-score**  **(35)** | ***p*-value** |
| **Sex (F/M)** | 12/10 | 8/7 |  |  |
| **Age (years)** | 23.4 (0.6) | 72.2 (1.9) |  |  |
| **Years of education** | 16.7 (0.4) | 15.4 (0.7) | 1.7 | ns |
| **Supermarket items**  **(correct answers)** | 24.1 (0.8) | 22.7 (0.9) | 1.6 | ns |
| **Body parts**  **(correct answers)** | 23.3 (0.8) | 22.9(0.9) | 0.4 | ns |
| **Countries**  **(correct answers)** | 21.7 (0.9) | 21.2 (0.8) | 0.4 | ns |
| ^a^ Mean values and standard errors in parentheses. Non-significant = ns. | | | | |
